# Social fear extinction susceptibility is associated with Microbiota-Gut-Brain axis alterations

**DOI:** 10.1016/j.bbi.2024.06.009

**Published:** 2024-06-08

**Authors:** Nathaniel L. Ritz, Thomaz F.S. Bastiaanssen, Caitlin S.M. Cowan, Linda Smith, Nigel Theune, Marta Brocka, Eibhlís M. Myers, Rachel D. Moloney, Gerard M. Moloney, Andrey N. Shkoporov, Lorraine A. Draper, Colin Hill, Timothy G. Dinan, David A. Slattery, John F. Cryan

**Affiliations:** a APC Microbiome Ireland, University College Cork, Cork T12YT20, Ireland; b Dept. of Anatomy and Neuroscience, University College Cork, Cork T12YT20, Ireland; c School of Microbiology, University College Cork, Cork, T12K8AF, Ireland; d Dept. of Psychiatry and Neurobehavioural Science, University College Cork, Cork T12YT20, Ireland; e Dept. of Psychiatry, Psychosomatics and Psychotherapy, University Hospital Frankfurt, Frankfurt 60528, Germany

**Keywords:** Microbiome, Social anxiety disorder, Social fear conditioning, Social phobia

## Abstract

Social anxiety disorder is a common psychiatric condition that severely affects quality of life of individuals and is a significant societal burden. Although many risk factors for social anxiety exist, it is currently unknown how social fear sensitivity manifests biologically. Furthermore, since some individuals are resilient and others are susceptible to social fear, it is important to interrogate the mechanisms underpinning individual response to social fear situations. The microbiota-gut-brain axis has been associated with social behaviour, has recently been linked with social anxiety disorder, and may serve as a therapeutic target for modulation. Here, we assess the potential of this axis to be linked with social fear extinction processes in a murine model of social anxiety disorder. To this end, we correlated differential social fear responses with microbiota composition, central gene expression, and immune responses. Our data provide evidence that microbiota variability is strongly correlated with alterations in social fear behaviour. Moreover, we identified altered gene candidates by amygdalar transcriptomics that are linked with social fear sensitivity. These include genes associated with social behaviour (*Armcx1, Fam69b, Kcnj9, Maoa, Serinc5, Slc6a17, Spata2*, and *Syngr1*), inflammation and immunity (*Cars, Ckmt1, Klf5, Maoa, Map3k12, Pex5, Serinc5, Sidt1, Spata2*), and microbe-host interaction (*Klf5, Map3k12, Serinc5, Sidt1*). Together, these data provide further evidence for a role of the microbiota-gut-brain axis in social fear responses.

## 1 Introduction

Social anxiety disorder (SAD) is a heterogeneous psychiatric condition defined by fear and avoidance of social scenarios due to the pre-conception of possible scrutiny by others (Sarmiento and Lau, 2020). Like other anxiety disorders, SAD can manifest in different intensities and symptoms. Although it is commonplace for many to experience fear or anxiety of specific social situations, individuals with SAD typically have a stronger response to negative stimuli that may trigger their anxiety in a manner they are unable to effectively cope with (Stein and Stein, 2008). Research into SAD is sparse compared to other psychiatric disorders and more specific therapeutics are needed that target the underlying neurobiology as opposed to minimizing the symptoms. While cognitive behavioural therapy has proven to be an effective option for long-term treatment of SAD there is a need for novel treatment directions and options (Baldwin et al., 2014).

The gut microbiota has been increasingly associated with social behaviour and has been shown to be capable of modulating a variety of social interactions across the animal kingdom (Sherwin et al., 2019, Pinacho-Guendulain et al., 2022). Previously, stress has been shown to incur social deficits restored by gut microbiota administration and specifically associated with *Enterococcus faecalis* introduction (Wu et al., 2021). Environmental stressors such as air pollution and maternal stress have also been shown to alter the microbiota in the offspring and induce social behaviour deficits (Smith et al., 2023). Resilience to chronic social defeat stress has been linked with corticotrophin-releasing factor expression in the brain and immune cells in circulation (Gururajan et al., 2019). Microbiota volatility, or stress-induced changes in the bacteriome composition over time, was elevated in individuals with higher stress markers in humans and mice (Bastiaanssen et al., 2021). Such volatility was also associated with resilience and susceptibility to behavioural response in a post-traumatic stress model (Tanelian et al., 2022). More recently, the virome has also been shown to be modified by chronic stress in the mouse (Ritz et al., 2024a) and has been unveiled as an understudied component of the microbiota in the context of stress and social-related behaviours.

Taken together, our current knowledge indicates that the microbiota is involved in social behaviour (Sherwin et al., 2019) and resilience to various stressors, suggesting that social fear response is also likely sensitive to microbiota composition. The microbiota-gut-brain axis, which describes the multidirectional influence of these systems on one another, is one such potential therapeutic target for SAD. Previously we have shown that the gut microbial community is altered in individuals with SAD (Butler et al., 2023) and that microbiota transfer from individuals with SAD to mice increases social fear sensitivity (Ritz et al., 2024b). However, the underlying etiology of how some individuals are predisposed to heightened social fear and anxiety is unclear, especially after a social stressor.

There is a wide range of individual responses to different stressors, and indeed stress resilience is a phenomenon driven by multiple systems, including the immune, endocrine, and central nervous systems (Feder et al., 2009, Bagot et al., 2016, Gururajan et al., 2019). To develop novel diagnostic and therapeutic options, it is advantageous to identify factors that are associated with social fear resilience and elimination in addition to understanding what may predispose some individuals to increased sensitivity and susceptibility to social anxiety. Transcriptomics of the hippocampus and amygdala, brain regions sensitive to stress and anxiety, further allows for deep gene expression analyses in the period following social fear stimulus and exposure. Herein, we have adapted the validated social fear conditioning paradigm (Toth et al., 2012, Toth et al., 2013), a preclinical murine model of SAD, to untreated (i.e., control) mice and stratified them dependent on their social behaviour measured during extinction to further explore the biological underpinnings of social fear.

## 2 Methods

### 2.1 Animals

This study used male C57Bl/6J mice (*N* = 48; 8 weeks of age on arrival; Envigo). Animals were habituated for 2 weeks after which treatments began. The holding room was under a 12-hour light/dark cycle (7:00–19:00), with a temperature of 21 °C and humidity of 10 %. Standard rodent chow (Teklad Global 18 % Protein Rodent Diet, sterilised) and water were available *ad libitum* throughout the experiments. To determine probable effect sizes for the behavioural readout as a consequence of social fear conditioning an examination of the literature, our publications, and in-house data were carried out. Procedures for the study took place over 3 days: on day 1, mice underwent social fear conditioning; on day 2, social fear extinction took place immediately followed by faecal pellet collection for microbiota analysis; on day 3, animals were euthanised by rapid decapitation and tissues were collected for secondary analyses. All tissues from all mice were included in the behavioural and molecular analyses. All procedures involving animals were conducted in accordance with the European Directive 2010/63/EC, the requirements of the S.I. No 543 of 2012 and approved by the Animal Experimentation Ethics Committee of University College Cork and the Health Products Regulatory Authority (HPRA AE19130 P118). Framework.

### 2.2 Social fear conditioning

**Fig 1:**
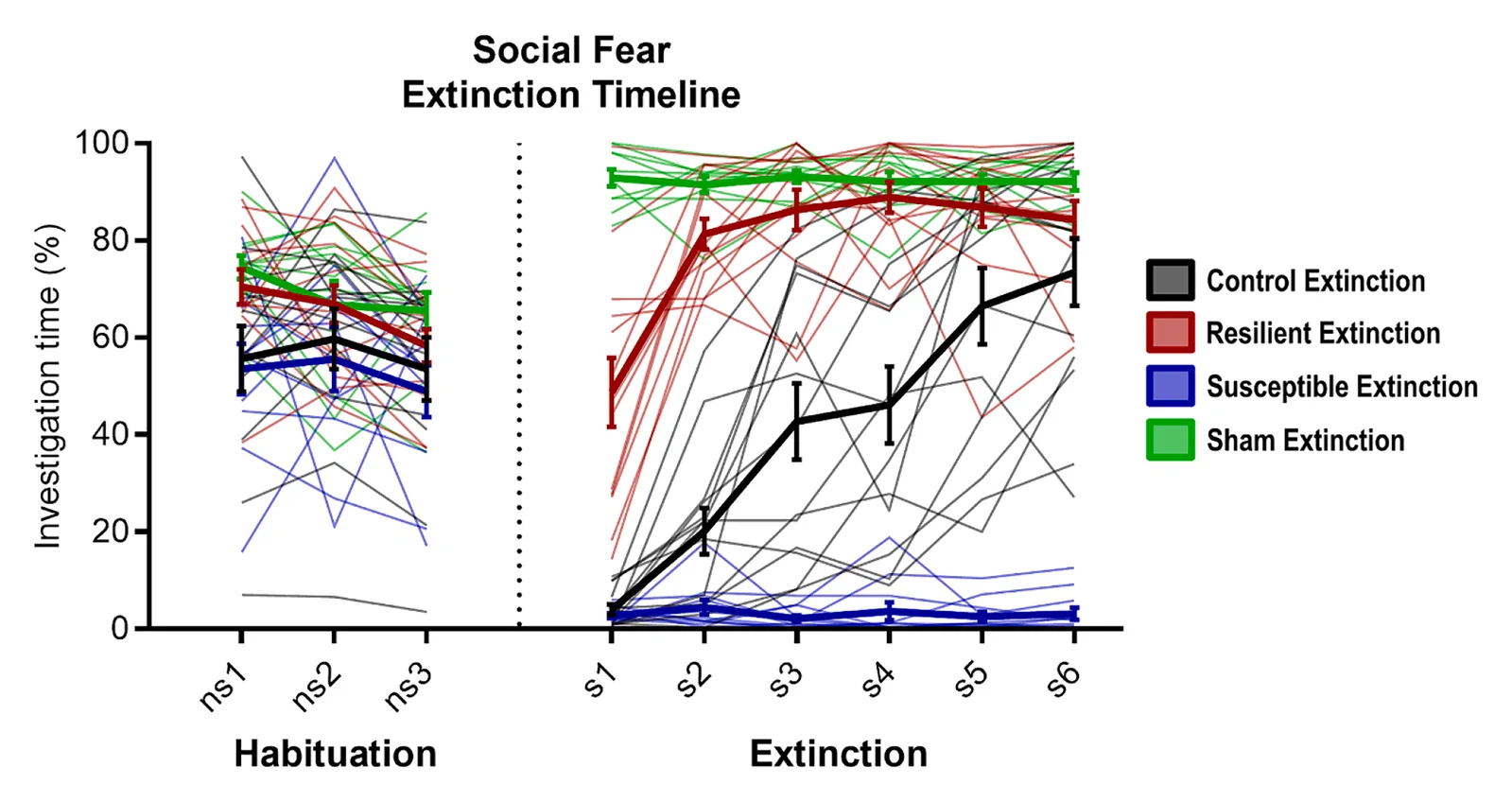
Social fear extinction behaviour response following social fear conditioning. Mice were stratified into three groups dependent on their social fear behaviour using a latent class linear mixed effects model. The sham group went through the same procedures but received no foot shock. The social fear extinction timeline shows the 3 non-social investigation trials (ns1-3; W(4) = 1.6982, *p* = 0.791) and the social investigation trials (s1-6; W(10) = 142.817, *p* < 0.001) with group differences tested using the linear mixed-effects model. Control Extinction *n* = 13, Resilient Extinction *n* = 13, Susceptible Extinction *n* = 11, and Sham Extinction *n* = 11; *p* < 0.05*, *p* < 0.001***.

Social fear learning and social fear extinction were assessed in the social fear conditioning (SFC) test (Fig.1f,g) (Toth et al., 2013). One week before SFC, mice were singly housed and continued to be for the remainder of the study. The SFC paradigm consisted of a single conditioning session on the first day, followed by a single extinction session on the following day. The conditioning chamber consisted of a transparent box (29x25x25cm) enclosed in a wooden chamber to reduce external noise and visual stimulation. The floor consisted of a removable stainless-steel grid connected to a shock delivery unit used for manual application of foot shocks. A video camera positioned above the chamber enabled recording.

During conditioning, mice were placed in the conditioning chamber with an empty wire mesh cage (10x8x7cm) placed in a corner as a nonsocial stimulus. Mice were allowed to freely explore the chamber and the non-social stimulus for 3 min, then it was replaced by an identical cage containing a novel conspecific (the social stimulus). After 2 s of interaction (defined by sniffing of the social stimulus), the experimental mouse was manually administered a 1 s electric foot shock (0.6 mA). This was repeated each time the experimental animal returned to interact with the social stimulus. When no further social contact was made for a 2 min period, mice were returned to their home cage. The first social contact and food shock occurred within 30 s for all experimental mice, and all mice received between 1- and 5-foot shocks over a maximum 5 min interval. Locomotor activity, as measured by distance traveled during habituation, and the time mice spent investigating the non-social stimulus, as a pre-conditioning measure of anxiety, were analysed using an automated tracking program (Ethovision 11.5, Noldus, the Netherlands). Wire mesh cages were cleaned with Trigene disinfectant between each extinction session.

On the next day, social fear extinction (SFE) was assessed in the home cage of each experimental mouse. There was a total of 9 trials in the extinction session, each consisting of a 3 min exposure to a social or non-social stimulus (placed on a short wall of the home cage), with a 3 min inter-trial interval. For the first 3 trials, experimental mice were exposed to three non-social stimuli identical to the cage used during conditioning. Then mice were exposed to six novel conspecifics (social stimuli) enclosed within the wire mesh cages. To reduce conditioned fear responses to the non-social stimulus, a clean wire mesh cage was placed in the home cage overnight between conditioning and extinction days. Wire mesh cages were cleaned with 70 % ethanol between each extinction session. Stimulus investigation, defined as time spent inter-acting within 7 cm of social stimulus, was analysed using an automated tracking program (Ethovision 11.5, Noldus, the Netherlands) and assessed as a readout of social fear during each trial of extinction.

The goal of this study was to analyze the innate variability in social fear extinction response of C57Bl/6 mice. We assessed the possibility of latent behavioural classes dependent on extinction response using a Bayesian approach. Stratification was determined using a latent class linear mixed effects model with Brownian motion modelling the correlation between trials (Proust-Lima et al., 2017). Using this technique mice were stratified into 3 distinct groups, Control Extinction (Con. Ext.), Resilient Extinction (Res. Ext.), or Susceptible Extinction (Sus. Ext.) dependent on their social investigatory behaviour measured during SFE. The Sham group was included to show baseline social approach behaviour to multiple conspecifics, but since the group did not experience conditioning no further measures were taken as their responsiveness to the social stress stimulus was undefined.

### 2.3 Corticosterone stress marker

Corticosterone quantification of plasma samples (10 µl) collected during euthanasia was performed using a corticosterone ELISA and conducted according to manufacturer’s guidelines (catalogue no. ADI-901–097, Enzo Life Sciences, Invitrogen, USA). Samples were analysed in duplicate in a single assay, the threshold detection was less than 32 pg/mL; coefficient of variation limit = 20 %; the concentration units are expressed in ng/mL. Light absorbance was read with a multi-mode plate reader (Synergy HT, BioTek Instruments, Inc., USA) at 405 nm.

### 2.4 Hypothalamic gene expression

The hypothalamus was dissected fresh and snap frozen for storage. Total RNA was extracted using the mirVANA kit (catalogue no. AM1561; Qiagen®, Germany) according to manufacturer’s instructions. RNA concentrations were quantified using a Nanodrop™ spectrophotometer (Thermofisher Scientific®, USA). Complementary DNA (cDNA) was then synthesized using High-Capacity cDNA Reverse Transcription Kit (catalogue no. 4368814; Thermofisher Scientific®).

Oxytocin receptor (*Oxtr*) and vasopressin receptor 1a (*Avpr1a*) genes were selected for analysis. Then RT-qPCR was performed using SYBR® Green real-time PCR on the cDNA samples using SYBR green (KAPA SYBR® FAST Kit; catalogue no. KK4602; Sigma, USA) to evaluate gene expression levels. Gene expression levels were then analysed on an AB7300 system (Applied Biosystems, Thermofisher®). Expression levels were calculated as the average of three replicates for each biological sample from Control Extinction, Resilient Extinction, and Susceptible Extinction groups (*n* = 11–13 per group) relative to β-actin expression. Then, fold changes were calculated using the 2^-ΔΔCT^ method (Livak and Schmittgen, 2001).

### 2.5 Transcriptomic sequencing

Hippocampus and amygdala RNA were extracted using the mirVANA kit (catalogue no. AM1561; Qiagen®, Germany). Source Bioscience conducted mRNA sequencing on the Illumina NovaSeq 6000 (S4 flow cell, 150 bp paired-end lane yielding 2.5 billion read pairs). Reference genome of obtained sequences was performed using the reference annotation: *Mus musculus* (organism), GRCm38, UCSC, genome browser (GRCm38.p6), Ensemble (Okazaki et al. 2002). Reads were assessed and filtered for quality using fastqc using default parameters. Genes were annotated and counted using kallisto using default parameters (Bray et al., 2016).

### 2.6 Processing of transcriptomics sequencing data

Further handling of transcriptomic data was undertaken in R (version 4.2.2) using the Rstudio GUI (version 2022.7.2.576). Principal component analysis was performed on centred log-ratio transformed (clr) values. Zeroes were replaced using the ‘const’ approach described by Lubbe and colleagues (Lubbe et al., 2021). Differences in overall gene expression was computed in terms of Aitchison distance (Euclidean distance between clr-transformed data) and assessed by PERMANOVA using the vegan package.

Differential gene expression was assessed using generalised linear models using the base R stats lm framework. Custom functions and scripts are available online at https://github.com/thomazbastiaanssen/Tjazi (Bastiaanssen et al., 2023a). Before statistical testing, gene counts were filtered by variance using the genefilter library in R (Bourgon et al., 2010), with 0.5 set as the cutoff.

### 2.7 Inflammatory cytokine analysis

Cytokine levels from plasma and splenocyte supernatant samples collected during euthanasia were quantified using the U-PLEX Custom Biomarker Group 1 (mouse) Assays (K15069M-2, Meso Scale Discovery, USA) measuring IFN-γ, TNF-α, IL-1β, IL-6, IL-10. Cytokine quantification was carried out according to the manufacturer’s guidelines. Samples below the detection limit of each assay were excluded. The lower threshold detection limits for each cytokine were: IFN-γ = 0.16 pg/mL, TNF-α =1.3 pg/mL, IL-1β= 3.1 pg/mL, IL-6= 4.8 pg/mL, and IL-10= 3.8 pg/mL.

### 2.8 Faecal bacteriome collection and sequencing

Murine faecal bacteriomes were analysed to assess the differences in community level composition across Control Extinction, Resilient Extinction, and Susceptible Extinction groups (the Sham Extinction group was excluded). Full metagenomic sequencing was conducted on faecal samples following social fear extinction.

A single faecal sample from each mouse per group (Control Extinction, Resilient Extinction, and Susceptible Extinction groups) was collected and used for analysis. The QIAamp Fast DNA Stool Mini Kit (Qiagen, Germany) was used to extract DNA from faecal pellets. This was performed following the manufacturer’s guidelines. Before extraction, however, 20 μL the ZymoBIOMICs spike-in control 1 (Zymo Research, USA) was added to aid in calculating absolute abundances. DNA concentrations were then measured using the Qubit High Sensitivity assay (Thermo Fisher Scientific, USA) before sequencing libraries were created using the Nextera XT Library Prep Kit (Illumina, USA). Library quality was assessed using the 4200 Agilent Tapestation (Agilent Technologies, USA) using the High Sensitivity D1000 ScreenTape assay (Agilent Technologies) before whole metagenomic sequencing was performed on an Illumina Novaseq 6000 platform using 2 × 150nt paired-end chemistry (Genewiz/Azenta, USA).

### 2.9 Faecal virome collection and sequencing

Murine faecal viromes were analysed to assess the differences in community level composition across Control Extinction, Resilient Extinction, and Susceptible Extinction groups (the Sham Extinction group was excluded). Full metagenomic sequencing was conducted on faecal samples following social fear extinction.

A single faecal sample from each mouse per group (Control Extinction, Resilient Extinction, and Susceptible Extinction groups) were collected and pooled in 650 µl of SM buffer. Fresh 0.5 M dithiothreitol was prepared in 1 mL of SM buffer. A volume of 16 µl of the dithiothreitol stock was added to samples to achieve a final concentration of 12 mM, and samples were incubated at 37 °C for 30 min. Centrifugation at 4,000g for 30 min at room temperature was used to pellet debris and bacterial cells. Subsequently, 400 µl of liquid was removed and treated with 40 µl of DNase/RNase buffer (50 mM MgCl2, 10 mM CaCl_2_), 12 µl (24U) of Turbo DNase (Thermo Fisher) and 4 µl (40U) of Fermentas RNase I (Fisher Scientific) at 37 °C for 1 h. Nucleases were inactivated by incubating at 65 °C for 10 min. With free nucleic acids now removed virus like particles (VLPs) were lysed using the QIAgen Blood and Tissue Kit following the manufacturer’s guidelines. However, an elution volume of 30 µl of Qiagen AE elution buffer (10 mM Tris-HCl, 0.5 mM EDTA, pH 9.0) was used to increase the final concentration of nucleic acid obtained.

Prior to library preparation, samples were adjusted to a volume of 52.5 μL sheared with a M220 Focused-Ultrasonicator (Covaris, Austria) applying the 350 bp DNA fragment length settings (peak power 50 W, duty factor 20 %, 200 cycles per burst, total duration of 65 s). Samples were then concentrated using the Genomic DNA Clean and Concentrator kit from Zymo Research and eluted in 16 µl. Library preparation was then carried out using xGen ssDNA and Low-Input DNA Library Prep Kit (Integrated DNA Technologies, USA) according to the manufacturer’s instructions. However, a final and additional bead clean-up step of 0.8 DNA/AMPure beads v/v ratio was included. Library QC to determine fragment length distribution and quantitation was performed using the 4200 Agilent Tapestation (Agilent Technologies, USA) using the High Sensitivity D1000 ScreenTape assay (Agilent Technologies) and Invitrogen Qubit. The normalised pooled library was then sequenced using 2 × 150 nucleotide paired-end sequencing chemistry on the Illumina NovaSeq 6000 platform at Genewiz (Leipzig, Germany).

### 2.10 Analysis of bacteriome sequencing data

Raw reads quality was assessed using FastQC (v0.11.8) (https://github.com/s-andrews/FastQC) and adapters were trimmed using Trimmomatic (v0.39) (https://github.com/usadellab/Trimmomatic) with parameters ILLUMINACLIP:/data/programs/trimmomatic/adapters/NexteraPE-PE.fa:2:30:10 SLIDINGWINDOW:4:20 MINLEN:60 HEAD-CROP:10 CROP:230. Trimmed reads were pooled and assembled using metaSPAdes (SPAdes v3.13.1) (https://github.com/ablab/spades) in paired-end metagenomic mode specified by parameters: −-meta. Assembled contigs from each extinction cohort were pooled and contigs < 1000 kb and redundant contigs were removed. Taxonomy and functional capacity of bacterial reads were generated using Woltka’s OGU classification pipeline (https://github.com/qiyunzhu/woltka/blob/master/doc/ogu.md). Analysis was done in RStudio following steps in the Bugs as Features pipeline (Bastiaanssen et al., 2023b; Bastiaanssen et al., 2023c).

### 2.11 Analysis of total viral sequences within the bulk metagenomic sequencing data

In addition to the contigs assembled by metaSPAdes in the bacteriome analysis, metaSPAdes was run again using the –metaviral parameter to ensure assembly of all viral contigs present. Viral contigs assembled from this step were pooled with contigs assembled in metagenomic mode and redundant sequences were removed. Contigs were identified as viral using VirSorter (version 2.2.3) following the viral sequence identification protocol (https://www.protocols.io/view/viral-sequence-identification-sop-with-virsorter2-5qpvoyqebg4o/v3). CheckV was used to assess the completeness of viral genomes. The final sequences were selected if they were circular (complete), deemed “high” in aai_completeness by CheckV or were filtered based on criteria recommended in aforementioned viral-id-sop pipeline where a contig is deemed potentially viral if viral_gene > 0, viral_gene = 0 AND (host_-gene = 0 OR score >=0.95 OR hallmark > 2). Viral read counts and coverage per sample was calculated using bowtie2 (version 2.4.2) A breadth of coverage filter was applied to the read counts, removing sequences with < 75 % coverage per sample. Viral contigs were aligned at nucleotide level using Blastn (v2.11.0 +) against an in-house Crass-virales database as well as the GVD, GPD, MGV, IMGVR and RefSeq viral databases (−evalue 1e-10). Viral sequences were translated using Prodigal (V2.6.3) with parameters −p meta and default translation table 11. Taxonomic classification of viral proteomes was assigned by Crassify (https://github.com/linda5mith/crassify).

## 3 Bioinformatics and statistical analysis of microbiome data

Data-handling of microbiome measures was undertaken in R (version 4.2.2) using the Rstudio GUI (version 2022.7.2.576). Principal component analysis was performed on centred log-ratio transformed (clr) values as a visual companion to the beta diversity analysis. Alpha-diversity metrics were calculated using scikit-bio (v0.5.8; https://scikit-bio.org/). For all analysis beyond alpha-diversity, taxa with a prevalence of < 5 % (at the species level for bacteria and vOTU level for viruses) were excluded from analysis as ratios are invariant to sub-setting and this study employs compositional data analysis techniques (Aitchison, 1982, Gloor et al., 2017). Zero values were then imputed for using the ‘const’ approach (Lubbe et al., 2021). Count data were then centred log-ratio (clr) transformed using scikit-bio. Differential abundance estimates were computed using the analysis of composition of microbiomes (ANCOM) (Mandal et al., 2015) method within scikit-bio. Beta diversity was computed in terms of Aitchison distance (Euclidean distance of clr-transformed counts) and differences in beta diversity were assessed using the PERMANOVA implementation from the vegan library using 1000 permutations (Aitchison et al., 2000). Plotting was handled using ggplot2 and the Python implementation of plotly (https://plotly.com/).

### 3.1 Statistics

Behavioural, immune, and gene expression data were assessed for outliers, defined as being outside of 2.5x standard deviations of the mean, and were subsequently excluded from analysis. Data was first assessed by a 1-way ANOVA across groups (Control Extinction, Resilient Extinction, Susceptible Extinction), then Tukey’s HSD post hoc analysis was used for pairwise comparisons. A linear mixed-effects model was used for repeated measures testing of non-social stimulus (ns1-3) and social stimulus (s1-6) social fear extinction trials. False discovery rate (FDR) analysis was performed using Storey’s FDR test of metagenomic and transcriptomic sequencing of differentially abundant taxa and expressed genes, respectively. Pearson’s correlation coefficient was used to measure associations between bacteriome or virome Aitchison’s beta diversity distance from the centroid and distance from mean social interaction score. Further statistical analysis was handled in R (v4.2.2) using the R Studio GUI (version 2022.7.2.576) and in Python with SciPy (v1.9.3). In all cases, an alpha threshold of 0.05 was used to denote significance.

## 4 Results

### 4.1 Social fear behaviour response undergoes typical, resilient, or susceptible extinction behaviours

Social fear conditioning (SFC) was conducted on all mice (*N* = 48) followed by social fear extinction (SFE) 24hr later. SFC, in brief, consisted of a habituation phase (exposure to the chamber environment), immediately followed by a conditioning phase where mice received an aversive stimulus (foot-shock exposure) upon interaction with a novel conspecific. The Sham Extinction group of mice underwent the same procedure but received no foot-shock upon social interaction (*n* = 11). The next day, during SFE, experimental mice were exposed to 9 home cage trials, each 3 min in duration with 3 min breaks between each trial. During the first 3 trials mice were exposed to an empty conspecific cage (non-social stimuli; ns1-3), then in the next 6 trials they were exposed to 6 novel conspecifics (social stimuli; s1-6; all different from the SFC conspecific) and the percent time spent investigating each stimulus was measured. Mice were then stratified into three different behavioural response groups dependent on their social behaviour using a latent class mixed effects model (Proust-Lima et al., 2017). Behaviour measured during social fear extinction showed no difference in the 3 non-social investigation trials (ns1-3; W(4) = 1.6982, *p* = 0.791), however, significant group effects were observed during the social investigation trials (s1-6; W(10) = 142.817, *p* < 0.001) (Fig. 1). Animals that underwent the expected gradient-based increase in social investigatory behaviour (Toth et al., 2012, Ritz et al., 2024b) over subsequent trials were placed in the group termed “Control Extinction” (Con. Ext.; *n* = 13). Animals that rapidly extinguished social fear were termed “Resilient Extinction” (Res. Ext.; *n* = 13) and animals that did not extinguish social fear were termed “Susceptible Extinction” (Sus. Ext.; *n* = 11). The Con. Ext. group was unique in this test in that they began with social fear behaviour indiscernible from the Sus. Ext. group in the first trial (s1), but then gradually improved over each subsequent trial until the end of the test where the Con. Ext. group did not differ statistically with the Res. Ext. group. The Res. Ext. group quickly abolished social fear by the second trial (s2) and then displayed a ceiling effect whereby on average they spent > 80 % of the time in social interaction akin to the sham SFC control group. The Sus. Ext. group did not increase the time spent in social interaction within the 6 social stimuli trials (Fig. 1).

Prior to SFC, mice were habituated to the conditioning chamber and locomotor activity and investigation time of the empty conspecific cages (non-social stimuli) were recorded. No differences were observed in locomotor activity as measured by total distance traveled during habituation (Fig. S1a). There also were no differences found in investigation time of the non-social stimuli (Fig. S1b). Last, the number of foot-shock exposures administered to inhibit repeated social approaches and interactions (minimum 1, maximum 5) was not different across groups (Fig. S1c). These data show that there were no measured inherent behavioural differences across the groups.

### 4.2 Social fear behaviour and bacteriome composition variability are positively associated

**Fig 2:**
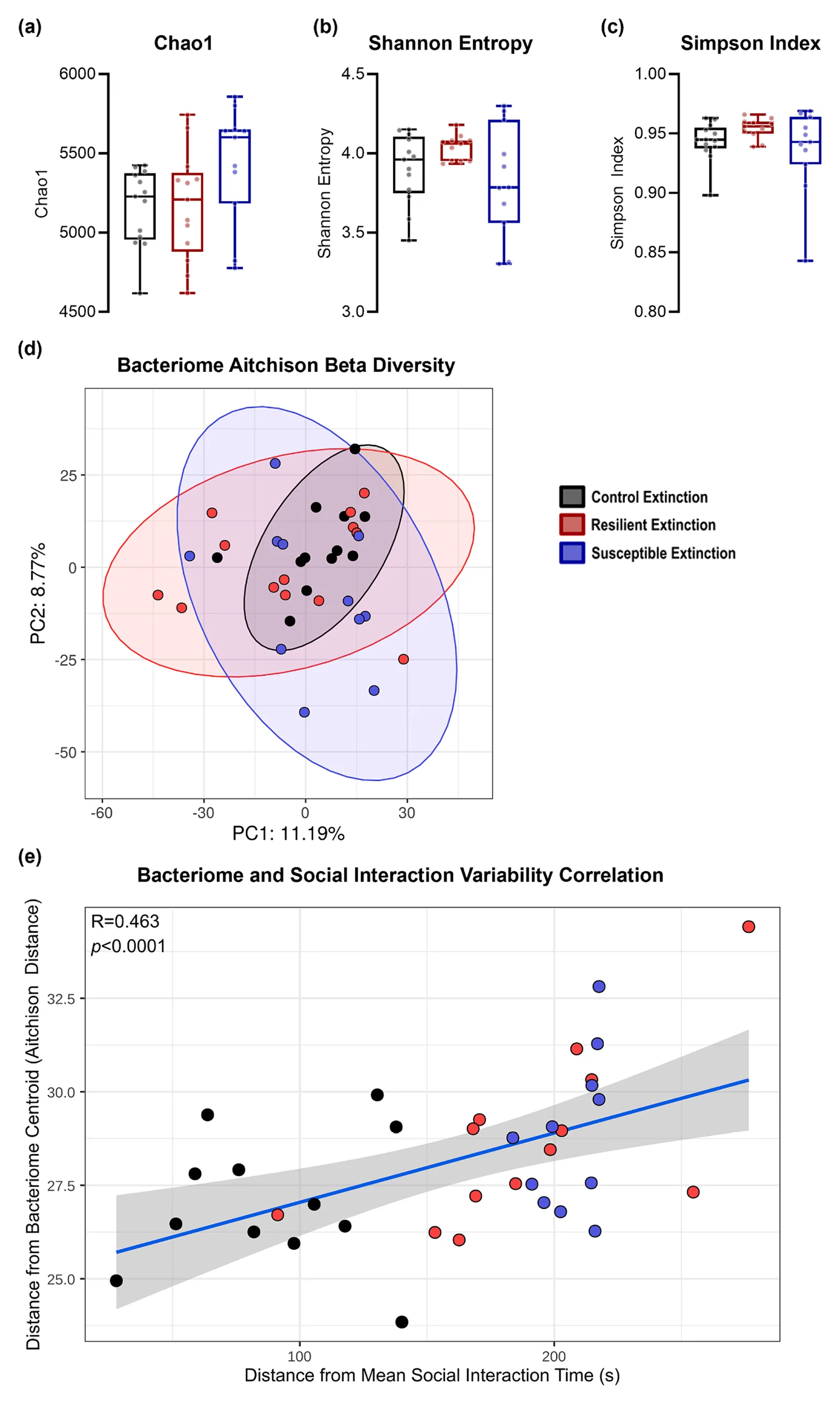
Social fear response and the bacteriome. **(a-c)** No statistical differences were found in bacteriome alpha diversity between groups. **(d)** No differences were found in bacteriome Aitchison beta diversity between groups. **(e)** There was a significant correlation (Pearson) between bacteriome distance from the centroid and social fear distance from the average total extinction (R = 0.463, t(34) = 3.64, *p* < 0.0001). Control Extinction *n* = 13, Resilient Extinction *n* = 13, and Susceptible Extinction *n* = 11.

**Fig 3:**
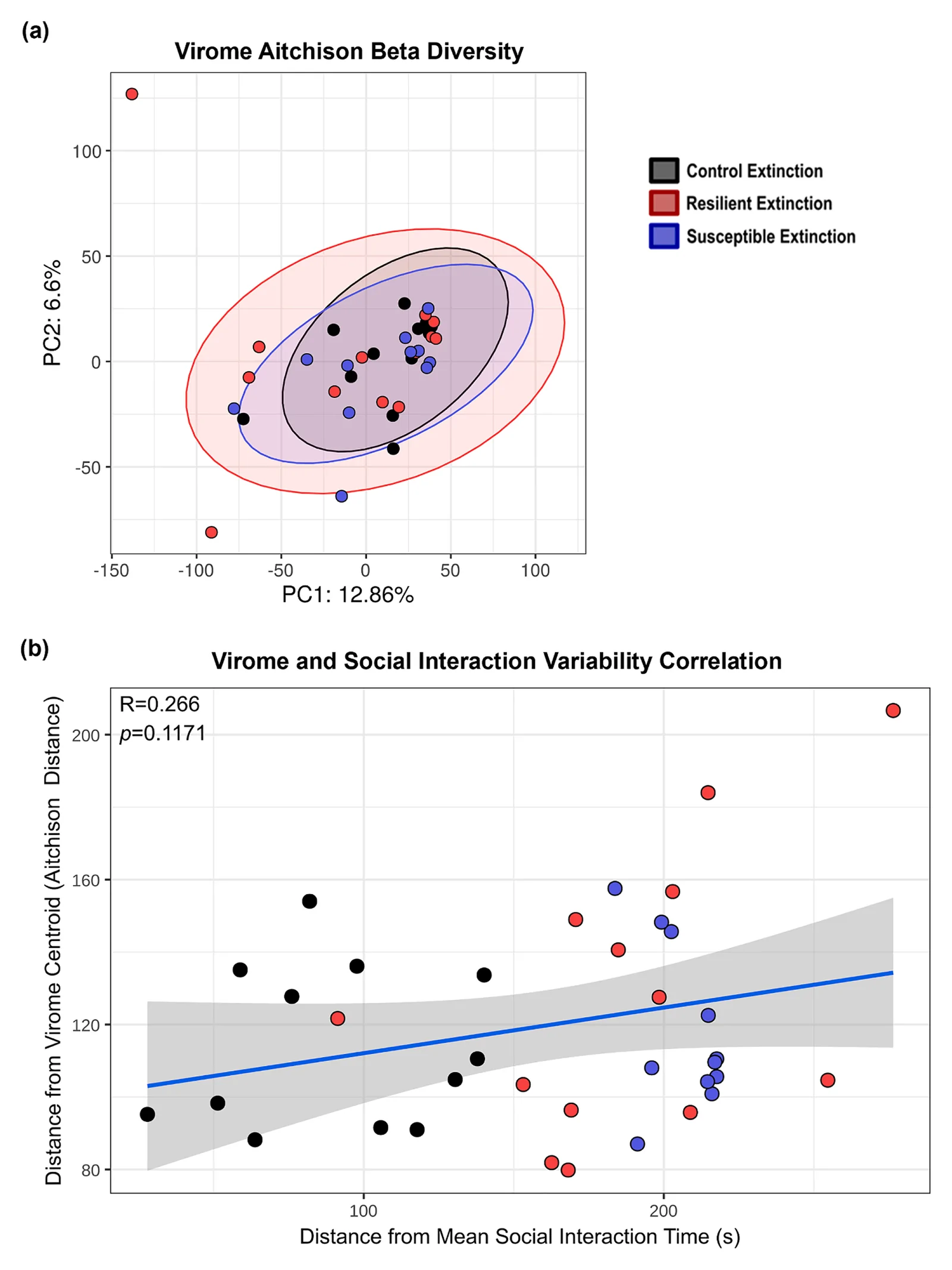
Social fear response and the virome. **(a)** No differences were found in virome Aitchison beta diversity between groups. **(b)** There was no statistical difference observed in the association between virome distance from the centroid and social fear distance from the average total extinction as measured by Pearson correlation (R = 0.266, t(34) = 3.64, *p* = 0.1171). Control Extinction *n* = 13, Resilient Extinction *n* = 13, and Susceptible Extinction *n* = 11.

The faecal bacteriome and virome were assessed by whole genome metagenomic sequencing collected after social fear extinction. No differences were observed in Chao1 (Fig 2a), Shannon Entropy (Fig. 2b) or Simpson Index (Fig. 2c). There was also no difference found in the bacteriome or virome Aitchison beta diversity distances between groups (Fig. 2d, 3a). However, we observed a strong correlation between bacteriome Aitchison distance from the centroid and distance from the mean total extinction social investigation time measured during SFE (R = 0.463, t(34) = 3.64, *p* < 0.0001; Fig. 2e). We also observed that the correlation between virome Aitchison distance from the centroid and distance from the mean total extinction social investigation time was similar to the bacteriome observation, however no significant difference was observed (R = 0. 266, t(34) =3.64, *p* = 0.1171; Fig. 3b). The correlation calculations were measured across all samples independent of group. These associations reveal a link between social fear behaviour and microbiome composition.

### 4.3 Group stratifications can be distinguished via differentially expressed genes in the amygdala

**Fig 4:**
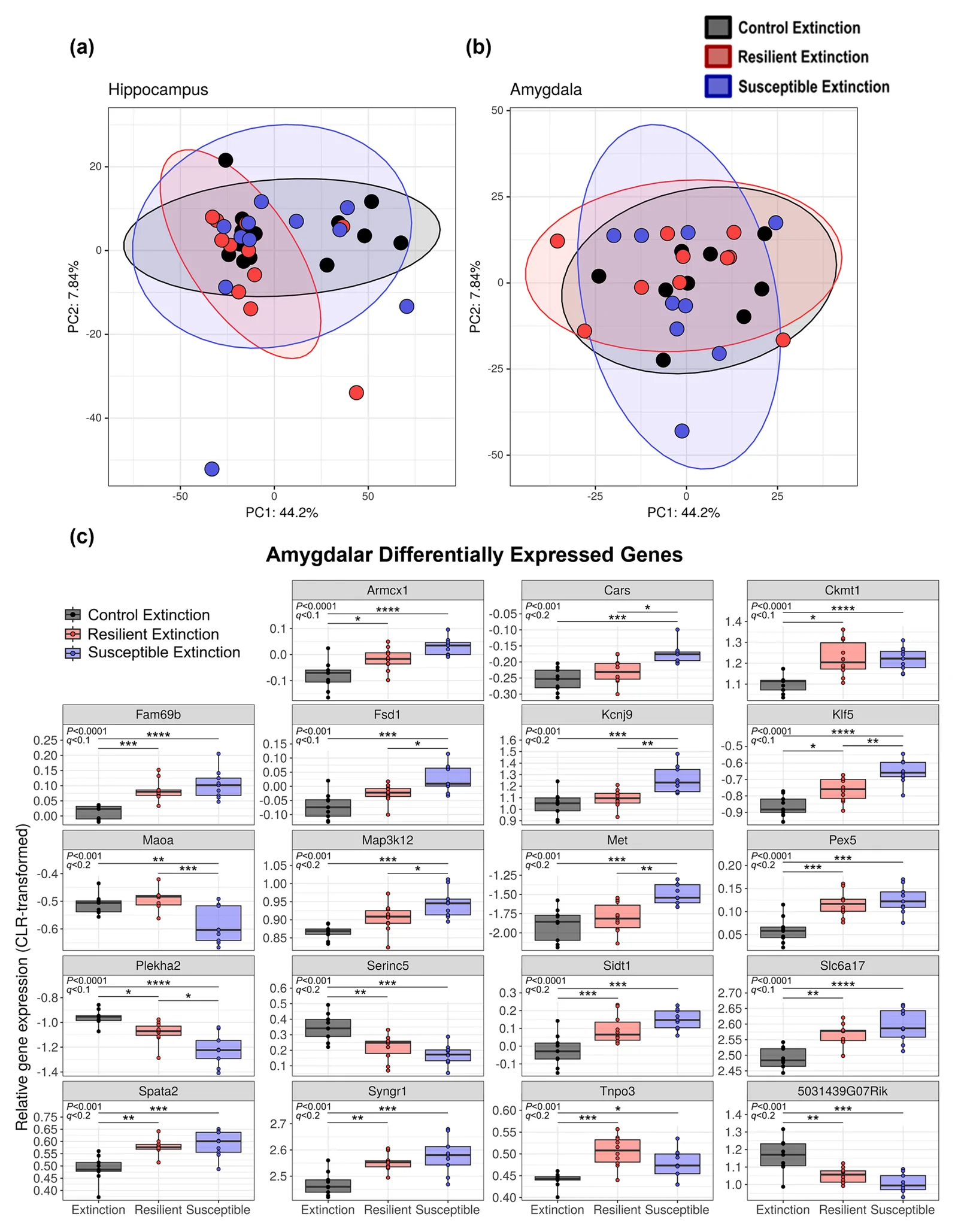
Differentially expressed genes in the amygdala between social fear extinction, resilient, and susceptible groups. **(a)** PCA of hippocampal gene expression. **(b)** PCA of amygdalar gene expression. **(c)** Differentially expressed genes in the amygdala (*P* = 1-way ANOVA. *q* = Storey’s FDR, *p* = pairwise Tukey’s HSD posthoc; *p* < 0.05*, *p* < 0.01**, *p* < 0.001***, *p* < 0.0001****). Control Extinction *n* = 13, Resilient Extinction *n* = 13, and Susceptible Extinction *n* = 11.

In order to understand the differences present in the brain at the transcriptional level, RNA sequencing was performed on the hippocampus and amygdala, collected 24hr after SFE. No group differences were found in principle coordinate analyses of each brain region (Fig. 4a, b). However, in the amygdala 19 genes were found to be altered across groups (Fig. 4c; statistics included in figure). These genes have been associated with social behaviour (*Armcx1, Fam69b, Kcnj9, Maoa, Serinc5, Slc6a17, Spata2*, and *Syngr1*), inflammation and immunity (*Cars, Ckmt1, Klf5, Maoa*, Map3k12, *Pex5, Serinc5, Sidt1, Spata2*), and microbe-host interaction (*Klf5*, Map3k12, *Serinc5, Sidt1*). These data reveal differences in amygdalar gene expression that accompany the social fear behaviour alterations across groups (accompanying statistics can be found in Fig. 4c).

### 4.4 No differences were observed in social and stress neuropeptide hormones and inflammatory cytokines

To narrow down the mechanism by which social fear responsivity occurs, we measured social and stress-related neuropeptides. Corticosterone was measured in basal-state plasma the day after SFE, however, there was no difference across groups (Fig. S2a). Then, we measured basal-state plasma oxytocin, but again there were no differences (Fig. S2b). Next, we measured expression of oxytocin and vasopressin receptors in the hypothalamus, a key region that both produces and is activated by these hormones, but we observed no differential effects (Fig. S2c, d). Last, we measured basal-state plasma inflammatory cytokines, but no differences were observed across groups in tumour necrosis factor alpha (TNF-α; Fig. S2e), interferon gamma (IFN-γ; Fig. S2f), or anti-inflammatory cytokine interleukin 10 (IL-10; Fig. S2g). The lack of effects in these data point away from oxytocin-related or corticosterone neuropeptide modulation or peripheral immune activation as the mechanisms driving social fear response.

## 5 Discussion

There is a growing body of literature implicating the host microbiota modulating the brain and behaviour (Johnson and Foster, 2018, Cryan et al., 2019, Morais et al., 2021), anxiety (De Palma et al., 2017), and especially the social brain (Sylvia and Demas, 2018, Sherwin et al., 2019, Sarkar et al., 2020, Wu et al., 2021). Here, we show that microbiota composition and amygdala gene expression correlate with social fear extinction behaviour following conditioning, in a murine model of SAD. These data further posit the microbiota-gut-brain axis as a key biological factor that is relevant to social fear responses and is a potential therapeutic target.

We found that mice could be stratified into three general social fear response categories: typical extinction, resilient, and susceptible. Mice that underwent typical extinction, high social fear in the beginning trials as defined by low social interaction time that gradually increased throughout the test, were placed in the “Control Extinction” group (Toth et al., 2012). Mice that had high social interaction time (low social fear) were termed “Resilient Extinction” and quickly extinguished their social fear. Lastly, mice that had low social interaction time throughout the test were termed “Susceptible Extinction”. Of note, there were no differences recorded in baseline locomotor and investigatory behaviours measured during the habituation phase of SFC (prior to any negative stimuli) nor any significant differences in the total number of foot-shock exposures per group. The Sham Extinction group went through an identical experimental approach but did not receive a foot-shock during conditioning. Sham Ext. data are included in the behavioural analysis to show the unconditioned social behavioural response of animals; however, they were excluded from subsequent analyses as their innate biological differences, which may determine their social fear conditioned response, are unknown and incomparable to the conditioned cohort despite their similar social approach behaviour under non-shocked conditions.

Somewhat surprisingly both the bacteriome and virome measured across the three social fear categorical groups were similar with no major deviations in alpha or beta diversities or the differential abundance of individual strains. Mice were single housed for one week prior to SFC to increase social approach, this also prevents microbiota transfer between co-housed animals prior to and during conditioning and extinction. However, we found an intriguing correlation between the difference from the mean social interaction total extinction scores versus the distance from average bacteriome composition (Aitchison distance from the bacteriome centroid). This correlation suggests the more divergent the bacteriome the further the individual social fear response will be from typical fear extinction. Interestingly, in the same analysis when looking at the virome, the data visually appears to move in a similar direction, but it did not reach statistical significance. This finding is similar to a previous finding by our group where the volatility of a microbiome, defined as Aitchison distance between longitudinal measures in a single individual (*i.e*., temporal variability), positively correlated with stress severity measures both in human and mice (Bastiaanssen et al., 2021). Together, these findings indicate that microbiota stability and centrality in a neutral and healthy setting are informative to mental health in terms of stress response and social fear behaviour.

To better understand how social fear response manifested at the transcriptional level in the brain we conducted RNA sequencing on the hippocampus and amygdala regions. These regions are responsive to stress, social interaction, fear, and learning and memory in addition to being sensitive to microbiota influence (Olsson and Phelps, 2007, Irle et al., 2010, Machado-de-Sousa et al., 2014, Zoicas et al., 2014, Qi et al., 2018, Sharvin et al., 2023). Furthermore, these regions were previously shown to contain differences in oxytocin receptor binding following SFC (Zoicas et al., 2014). The hippocampus displayed few changes across groups in this study and did not survive false-discovery rate analyses, however the amygdala had 19 genes that were differentially expressed following SFC, extinction, and behaviour-based stratification.

Further assessment of the differentially expressed amygdalar genes can shed light on potential transcriptomic factors that affect social fear resiliency and susceptibility. Armadillo repeat containing X-linked 1 (*Armcx1*) was downregulated in control animals that underwent extinction in comparison to the other groups. The *Armcx1* region overlaps with the quantitative trait loci of Memory quantitative locus 13 (*Memor13*) and 14 (*Memor14*), both of which have been implicated in social behaviour in rats (Ruiz-Opazo and Tonkiss, 2006). Potassium inwardly rectifying channel subfamily J member 9 (*Kcnj9*) was upregulated in extinction susceptible animals and has been identified as a risk locus in psychiatric disorders (Gunturkun et al., 2022). *Kcnj9* (also known as *Girk3*) has previously been linked with incentive salience and drug reward behaviour (Herman et al., 2015). Divergent Protein kinase domain 1b (*Fam69b*), reduced in the control extinction animals, localises to the endoplasmic reticulum (Tennant-Eyles et al., 2011) and has been implicated in neurological disorders (Dudkiewicz et al., 2013). Monoamine oxidase A (*Maoa*) was significantly reduced in extinction susceptible animals and has been shown to be altered in human anti-social behaviour following previous exposure to abuse (Fergusson et al., 2011, Fergusson et al., 2012). Interestingly, *Maoa* functional connectivity has also been associated with psychosocial stress response in the human brain (Sun et al., 2020). Serine incorporator 5 (*Serinc5*) was reduced in expression in the extinction resilient and susceptible animals compared to those in the control extinction group. *Serinc5* is involved in myelination and a reduction in which has been observed following a lack of social interaction in psychiatric disorders (Lubke et al., 2014). Solute carrier family 6 member 17 (*Slc6a17*) was upregulated in the extinction resilient and susceptible animals. *Slc6a17* mutations in humans have been shown to cause speech impairment and behavioural problems (Iqbal et al., 2015). Spermatogenesis associated 2 (*Spata2*) is an immune-related gene that was also upregulated in the extinction resilient and susceptible animals and has been associated with autistic traits (Arenella et al., 2022). The transcript for Synaptogyrin 1 (*Syngr1*) was also upregulated in the extinction resilient and susceptible animals and has been identified to undergo altered expression in patients with depression (Bagot et al., 2016), schizophrenia, and bipolar disorder (Verma et al., 2005). Furthermore, Creatine kinase mitochondrial 1 (*Ckmt1*) expression was downregulated in the control extinction animals, *Armcx1* and Cysteine tRNA synthetase (*Cars*) both had upregulated expression in the extinction susceptible group, while *Maoa* was downregulated in the extinction susceptible group, and interestingly these genes have been associated with mitochondrial function which can play a role in neuropsychiatric diseases (Datler et al., 2014, Cartoni et al., 2016, Pei and Wallace, 2018, Li and Yang, 2021).

There were also amygdalar genes associated with inflammation and immunity as well as host-microbe interaction. For example, Krupple-like factor (such as *Klf5*), which was increased in extinction susceptible animals and reduced in control animals, contributes to numerous conditions such as cerebrovascular, neurodegenerative, and cardiovascular diseases as well as pro-inflammatory states, and has been shown to mediate immunity to both bacterial and viral pathogen challenges (McConnell et al., 2008, McConnell and Yang, 2010, Yin et al., 2015, Wang et al., 2016). *Maoa*, reduced in the extinction susceptible animals, has been implicated as a marker for monocytes and macrophages (Cathcart and Bhattacharjee, 2014) capable of regulating psychosocial behaviour and stress response (Sun et al., 2020, Mentis et al., 2021). Mitogen-activated protein kinase kinase kinase 12 (Map3k12), increased in extinction susceptible animals, has been shown to modulate neuronal injury and neurodegeneration and also modulate antiviral immunity (Siu et al., 2018, Guan et al., 2023). Peroxisomal biogenesis factor 5 (*Pex5*), reduced in animals that underwent control extinction, has been linked with inflammation and Nuclear factor-κB (NF-κB) regulation in innate immune signalling response to bacteria and viruses (Dixit et al., 2010, Di Cara et al., 2017, Vijayan et al., 2017). *Serinc5*, increased in control extinction animals, has also been identified as an antiviral factor in addition to being associated with borderline personality disorder (Lubke et al., 2014, Timilsina and Stavrou, 2023). Systemic RNAi-defective transmembrane family member 1 (*Sidt1*), also reduced in control extinction animals, has been shown to play a role in interferon-1 response and associated with antiviral activity (Nguyen et al., 2019, Morell et al., 2022). Lastly, *Spata2*, reduced in control extinction animals, is involved in tumour necrosis factor (TNF) and NF-κB signalling modulating inflammation (Ren et al., 2021, Masola et al., 2022). TNF-α was found to be significantly reduced in the medial amygdala in mice that received human SAD microbiota for 4 weeks (Ritz et al., 2024b). The differential involvement of these genes along with the microbiota variability observed in social fear response suggests that complex microbiota-gut-brain axis interactions may underly the development of the social anxiety phenotype.

In tandem with untargeted metagenomics and transcriptomics, we also employed targeted biomarker assays to potentially link the susceptibility to the social fear behavioural phenotype with known hormones associated with social behaviour, anxiety, and previously observed in a murine SAD microbiota transfer model (Ritz et al., 2024b). Corticosterone and oxytocin have been shown to be modulated in social stress models and can affect social behaviour (Babb et al., 2014, Harvey et al., 2019) and intracerebroventricular administration of oxytocin receptor antagonist has previously been reported to attenuate social preference (Lukas et al., 2011) and social fear conditioning (Zoicas et al., 2014). These findings suggest involvement of neuroendocrine hormones in social behaviour. Therefore, we explored whether social or stress hormones were altered between our group designations but found no significant effects. There were no differences across groups in basal plasma oxytocin, or oxytocin or vasopressin receptor gene expression in the hypothalamus. Basal plasma corticosterone and circulating cytokines were similarly unchanged across groups. Social behaviour has been shown to be regulated by oxytocin (Donaldson and Young, 2008), which is particularly sensitive in the dorsolateral septum and has previously been shown to modulate social fear (Zoicas et al., 2014). We did not observe any altered oxytocin-related genes in the hippocampal and amygdalar transcriptomic dataset. These data suggest that an oxytocin neuroendocrine mechanism underlying the differential social response is unlikely mediated in the hippocampus and amygdala, but rather, governed by the hypothalamus and septum (Zoicas et al., 2014). Moreover, in our previous study where we transferred faecal microbiota from patients with SAD to mice, we found alterations in basal corticosterone and neuronal oxytocin (Ritz et al., 2024b). Since similar alterations were not observed herein, it posits that a longer-term social fear phenotype is necessary to observe broad neuroendocrine changes. Future research is necessary to further elucidate the underlying mechanism driving the connection between social fear response and the microbiota-gut-brain axis.

Moving forward there are questions that need to be further developed and elucidated. Sex differences are an important part of the social anxiety disorder story and the field at large. Females are more commonly afflicted with SAD than males (Asher et al., 2017), however, female mice have not been characterized to the same extent with the social fear conditioning behaviour model and the mouse strain used herein and will require further development of adequate tools and approaches to compensate for the inherent differences in murine sex differences (An et al., 2011, Toth et al., 2012, Menon et al., 2018). Therefore, we performed experiments with male C57Bl/6J mice to both ensure the effectiveness of the model and reduce statistical covariates. Moreover, microbiota sex differences were not found in our previous human social anxiety disorder studies (Butler et al., 2022, Ritz et al., 2024b). While this does not mean sex differences are marginal, we infer that the direct contribution of the microbiota to social fear is not the driving force behind social anxiety disorder-related sex differences. Another consideration is that single housing is a stressor in and of itself (Manouze et al., 2019). However, single housing is required in the social fear conditioning paradigm (Toth et al., 2012, Toth et al., 2013) to both increase social drive and social approach during conditioning. Furthermore, it was evenly applied to all animals in the study and thus uniformly controlled. Interestingly, differential expression of transcriptional data indicate that the control extinction group is more different to the resilient and susceptible extinction groups. One potential explanation for this finding is that the extinction group displayed temporal and contextual learning (the stepwise extinction of social fear) unlike the resilient and susceptible groups which showed ceiling and floor effects, respectively, on their ability to extinguish social fear across trials and this could be a result of the neuroplasticity of learning (McClung and Nestler, 2008, Akil and Nestler, 2023).

Social fear is a multifactorial behaviour that can be attributed to genetic, environmental, and host perception of social contexts. Despite these many factors, there is a relatively narrow diagnostic and treatment approach for individuals with SAD at present. Here we provide evidence in an empirical social fear model that extinction of social fear is variable, even in a controlled environment with a genetically identical single-housed strain of mice. We explored the potential for microbiota-related variability to be associated with social fear responsivity and found that microbiota variability strongly correlated with categorical extinction behaviour. These findings suggest that further research into microbiota-based diagnostics and treatments may provide novel therapeutic potential for the alleviation of SAD symptoms.

## Supplementary Material

**Appendix A. Supplementary data**

Supplementary data to this article can be found online at https://doi.org/10.1016/j.bbi.2024.06.009.

Supplementary Data 1

## Data Availability

Data will be made available on request.
